# Comprehensive analysis of research related to rehabilitation and COVID-19, hotspots, mapping, thematic evolution, trending topics, and future directions

**DOI:** 10.1186/s40001-023-01402-1

**Published:** 2023-10-13

**Authors:** Siddig Ibrahim Abdelwahab, Manal Mohamed Elhassan Taha, Monira I. Aldhahi

**Affiliations:** 1 Medical Research Centre, Jazan University, Jazan, Saudi Arabia; 2 Substance Abuse and Toxicology Research Centre, Jazan University, Jazan, Saudi Arabia; 3 Department of Rehabilitation Sciences, College of Health and Rehabilitation Sciences, Princess Nourah bint Abdulrahman University (PNU), P.O. Box 84428, 11671 Riyadh, Saudi Arabia

**Keywords:** COVID-19, Rehabilitation, Bibliometrics, Mapping, Knowledge structure, Thematic evolution

## Abstract

**Background:**

This study conducted a comprehensive analysis of research pertaining to the intersection of rehabilitation and COVID-19 (COV-REH). The main aim of this study is to analyze the thematic progression and hotspots, detect emerging topics, and suggest possible future research directions in the COV-REH.

**Methods:**

Appropriate keywords were selected based on the Medical Subject Headings (MeSH) PubMed database and the Scopus database were used to retrieve a total of 3746 original studies conducted in the English language. The data extraction was performed on June 30, 2023. VOSviewer and Bibliometrix utilize CVS and BibTex files to facilitate the performance analysis and generate visual maps. The performance indicators reported for the research components of the COV-REH were compiled using the Scopus Analytics tool.

**Results:**

From 2003 to 2023, 3470 authors from 160 organizations in 119 countries generated 3764 original research documents, with an annual growth of 53.73%. 1467 sources identified these scholarly works. Vitacca, M. (Italy), Harvard University (USA), and the USA published the most articles. This study included 54.1% of medical scholars. Telerehabilitation, exercise, quality of life, case reports, anxiety, and pulmonary rehabilitation were the primary themes of the COV-REH. One component of “telerehabilitation” is now the cardiac rehabilitation cluster. The trending topics in COV-REH are “symptoms,” “protocol,” and “community-based rehabilitation”.

**Conclusions:**

This study proposed several significant research directions based on the current thematic map and its evolution. Given that COV-REH investigations have been determined to be multidisciplinary, this study contributes conceptually to several fields and has wide-ranging implications for practitioners and policymakers.

## Contributions to the literature

- Conducted comprehensive analysis of rehabilitation and COVID-19 intersection (COV-REH).
- Explored thematic progression, emerging topics, and research directions using Scopus database (3746 studies).
- Utilized VOSviewer, Bibliometrix, and Scopus Analytics for performance analysis and visualization
- Provides directions for future research based on thematic map evolution.
- Proposed future research pathways based on evolving thematic map; multidisciplinary relevance for practitioners and policymakers.
- Revealed dominant themes: telerehabilitation, exercise, quality of life, anxiety, and pulmonary rehabilitation

## Background

COVID-19, also known as coronavirus disease 2019, is an infectious disease that is primarily caused by the SARS-CoV-2 virus [1]. The contagious nature of this phenomenon has facilitated the rapid spread of the disease. The COVID-19 pandemic has resulted in a significant loss of life, with a mortality rate exceeding one million individuals in the USA [2]. COVID-19 primarily induces respiratory manifestations that resemble common cold, influenza, or pneumonia. COVID-19 has the potential to affect not only the lungs and respiratory system but also other bodily systems. The disease may also affect other bodily regions [3, 4]. The majority of individuals afflicted with COVID-19 experience mild symptoms, while a subset of individuals may develop severe illness. Certain individuals, including those with mild or asymptomatic manifestations, may experience the development of post-COVID conditions, commonly referred to as “Long COVID.” COVID-19 is transmitted through the exhalation of respiratory droplets and aerosols containing viral particles by an infected individual. These droplets and particles have the potential to be inhaled by individuals or come into contact with their eyes, nose, or mouth. Under certain conditions, these droplets have the potential to contaminate the surfaces upon contact [5–8].

Research pertaining to the intersection of rehabilitation and COVID-19 (COV-REH) has gained significant attention due to the long-term effects of the virus on individuals’ physical and mental well-being [9, 10]. This area of investigation aims to understand and address the rehabilitation needs of COVID-19 patients, including those who have experienced severe illness or prolonged hospitalization [11, 12]. Studies in this field explore various aspects, such as respiratory therapy, physical rehabilitation, cognitive rehabilitation, and mental health interventions [9–11, 13–16]. Researchers investigate the effectiveness of different rehabilitation strategies, develop protocols, and evaluate outcomes to optimize recovery and enhance the quality of life for COVID-19 survivors. This research also focuses on identifying risk factors for functional impairments, understanding the impact of COVID-19 on distinct populations (such as older adults or individuals with pre-existing conditions), and designing targeted rehabilitation interventions [17, 18]. By advancing our knowledge in this area, research in COV-REH contributes to developing evidence-based practices and interventions for optimal recovery and long-term health outcomes.

The global impact of the COVID-19 pandemic has resulted in substantial obstacles for both rehabilitation services and research. The COVID-19 pandemic has significantly affected the provision of rehabilitation services, resulting in consistent disruption of essential health services [7, 8]. Simultaneously, the COVID-19 pandemic escalated the demand for rehabilitation services. This demand arises from two distinct groups: individuals who are severely afflicted by the disease and require intensive care, as well as those who endure persistent health complications stemming from the virus [10, 12, 17]. The demand for rehabilitation services has witnessed a notable surge amidst the ongoing pandemic, consequently leading to an accelerated pace of research in the field of COV-REH. To date, no thematic study has been conducted in the field of COV-REH. Notably, during the period following the COVID-19 outbreak, potential research directions have not been examined. Regrettably, no comprehensive quantitative study has been conducted to examine the significance of COV-REH, despite its pivotal role in the COV-REH revolution.

Bibliometric analysis is the most suitable tool for condensing copious amounts of data when confronted with a wide-ranging review and an extensive dataset that cannot be feasibly reviewed manually. This analysis allows the presentation of the intellectual framework and emerging patterns within a specific research theme in a given field [19]. This study employed bibliometric analysis as a methodological approach to systematically evaluate and quantify the existing body of research pertaining to the topic of COV-REH. An additional advantage of bibliometric analysis is its applicability to studies that encompass multiple disciplines, theories, and methodologies. The utilization of this methodology has experienced significant growth in the medical field over the past two decades, leading to the generation of novel insights [20, 21]. Bibliometric analysis facilitates the examination of the development of author keywords, authors, collaborations, and thematic trends within a specific field using bibliographic data.

Bibliometrics is a statistical approach that examines the attributes of publications and aims to quantify, describe, and forecast the scientific discourse processes. Over the course of time, studies on conversation have unveiled the behavior models and academic patterns that have been established within a particular field [22–24]. Therefore, bibliometric analysis can be used to examine performance, which specifically emphasizes productivity and the impact of publications in the field. The objective of this bibliometric analysis is to rectify the shortcomings by systematically organizing the scholarly achievements related to COV-REH. This study provides a comprehensive overview of the current fragmented literature and proposes directions for future research.

## Methods

### Study design

**Fig. 1:**
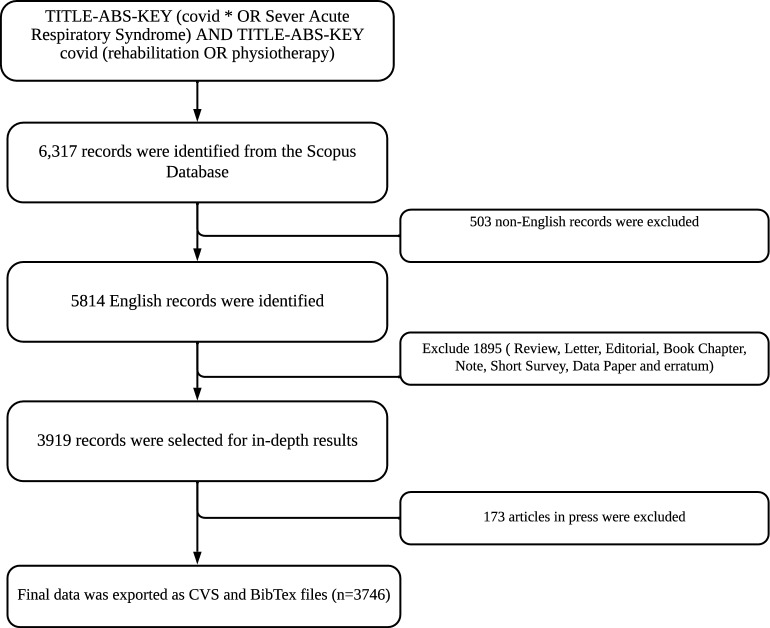
Study flow diagram of the search strategy in the database

Following the establishment of the research objective, target population, and methodology, a set of keywords was identified to conduct database searches. To ensure comprehensive coverage and minimize the risk of overlooking any articles, the dataset was augmented by incorporating the widely utilized Scopus database. Scopus, a database provided by Elsevier, holds the distinction of being the most extensive collection of abstracts and citations in scholarly literature that has undergone rigorous peer review. The Scopus database is widely globally recognized as the foremost citation database. The database comprises a comprehensive collection of scholarly articles sourced from globally recognized high-impact journals, encompassing open-access journals, conference proceedings, and books [25, 26]. Appropriate keywords for this study were selected based on Medical Subject Headings (https://www.ncbi.nlm.nih.gov/mesh/). The coverage of certain titles extends as far back as the year 2003. The database queries were conducted on June 30, 2023. The search strings used were ( TITLE-ABS-KEY ( covid* OR “severe acute respiratory syndrome” ) AND TITLE-ABS-KEY ( rehabilitation OR physiotherapy ) ) AND ( EXCLUDE ( PUBSTAGE, “aip” ) ) AND ( LIMIT-TO ( DOCTYPE, “ar” ) ) AND ( LIMIT-TO ( LANGUAGE, “English” ) ). The flow of the study is shown in Fig. 1.

### Exclusion and inclusion criteria and sample size

The study’s inclusion criteria encompassed original research articles that were published in the English language, indexed by Scopus, and released within the time frame of 2003–2023. Exclusions were made for articles published in languages other than English, as well as review papers, notes, letters to the editor, errata, books, or chapters. All articles in the press were excluded. According to the findings of previous bibliographic studies, it has been suggested that a sample size consisting of fewer than 200 bibliographic documents is insufficient for generating computational recommendations or conducting a dependable lexical analysis. This study used a sample size of 3746 research documents.

### Mapping and analysis

VOSviewer and Bibliometrix employ CVS and BibTex, respectively, files for the purpose of conducting performance analysis and generating visual maps. VOSviewer and Bibliometrix were employed previously in many researches [27–30]. The Scopus Analytics tool was utilized to compile the performance indicator report for the research components of the COV-REH. This study focused of this study revolved around the documents with the highest number of citations and the researchers, institutes, countries, and sources with the most publications. The study of thematic evolution involved the categorization of various clusters of research topics, supplemented by an analysis of the keywords used by the authors. The identification of trending topics was facilitated through the utilization of Bibliometrix, a tool that employs an analysis of the frequency and dynamics of authors’ keywords. The study’s time frame was partitioned into two distinct periods to ascertain thematic maps, thereby facilitating the identification of research topics that emerged within each respective period. This inquiry pertains to the emergence of new branches or merging of existing topics within the realm of research. This study also examined maps depicting the extent of international and local collaboration among researchers, institutes, and countries. Additionally, it aimed to distinguish between nations based on the level of exclusivity in their corresponding relationships.

## Results

From 2003 to 2023, a total of 3764 original research documents pertaining to COV-REH were produced by 3470 authors affiliated with 160 organizations across 119 countries (Table 1). A total of 1467 sources revealed the existence of these scholarly productions. The annual growth rate has reached 53.73%, with an average document age of 1.3. This finding aligns with prior research that has demonstrated the progression of studies pertaining to COVID-19. The volume of research in the years 2020–2023 is approximately 99%. 2022 and 2021 are the years in which research was conducted, estimated at two-thirds of the knowledge volume carried out in COV-REH. Research output from 2020 to 2023 exhibited a voluminous proportion of approximately 99%. The years 2022 and 2021 mark the period during which research was conducted, accounting for approximately two-thirds of the knowledge volume related to COV-REH.

**Table 1:** Main information about data

Timespan	2003–2023
Sources (Journals, Books, etc.)	1467
Documents	3746
Organizations	160
Countries	119
Annual Growth Rate %	53.73
Document Average Age	1.3
Average citations per doc	7.598
Keywords Plus (ID)	12,317
Author’s Keywords (DE)	6134
Authors	3470
Authors of single-authored docs	103
Authors Collaboration	
Single-authored Documents	108
Co-Authors per Document	7.47
International co-authorships %	18.8

**Fig. 2:**
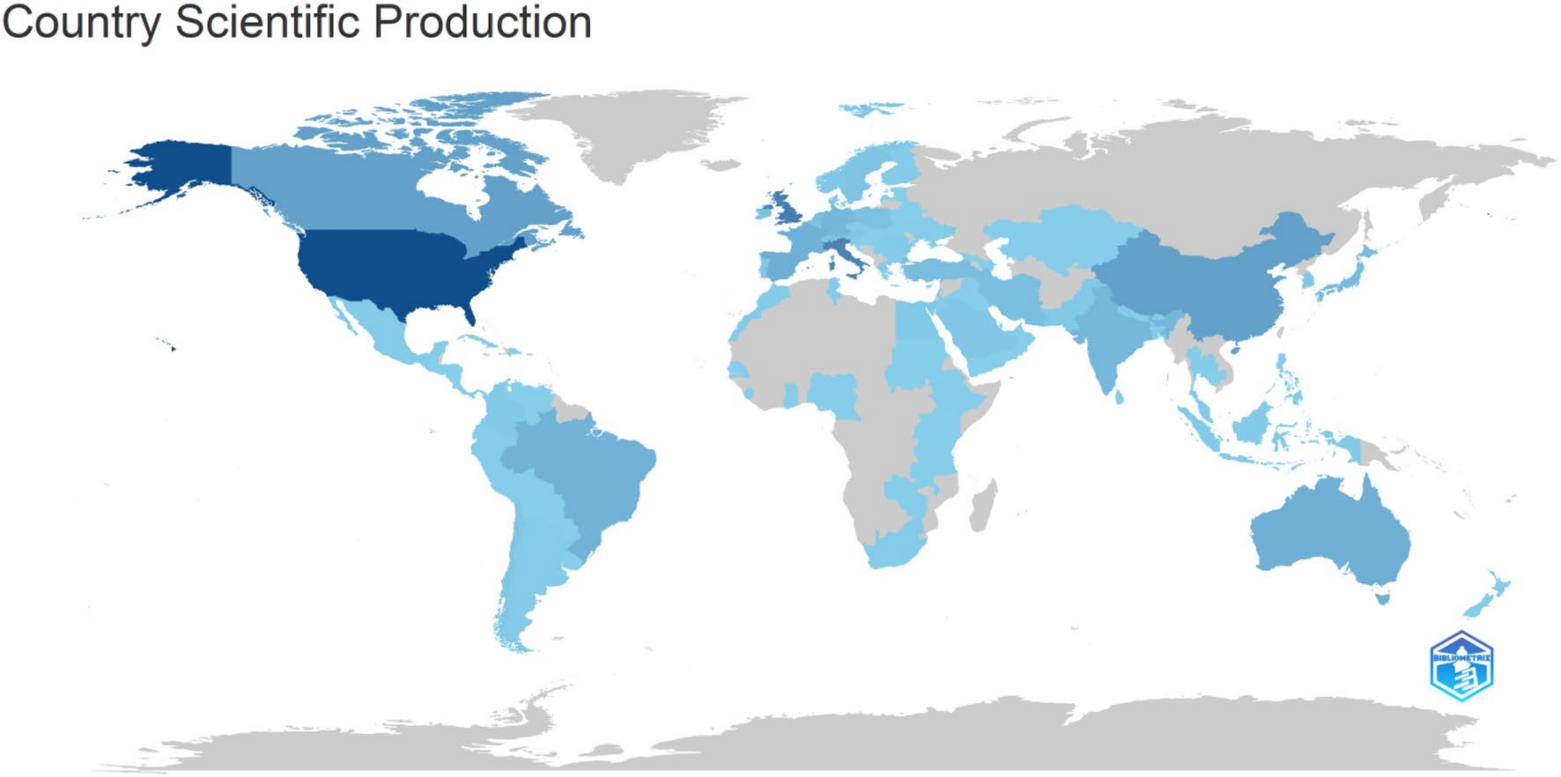
Country specific production in COV-REH. The density of the clue color indicates high production of COV-REH-related publications

Vitacca, M. of the Respiratory Rehabilitation Institute in Lumezzane, Italy, authored a total of 14 research articles, securing the top position. Research conducted by Vitacca primarily focused on three key areas: pulmonary rehabilitation, telerehabilitation, and the neuropsychological characteristics exhibited by individuals during their recovery from COVID-19 [16, 31–36]. The following is Sivan M. from the University of Leeds, UK, with 13 publications. Sivan and his research group have conducted a study on the development of an integrated rehabilitation pathway. Italy proposed an evidence-based report outlining an integrated rehabilitative approach for individuals with COVID-19. Remarkably, among the 119 nations, the USA (*n* = 834) exhibited the highest level of productivity, followed by the UK (*n* = 455), Italy (*n* = 421), China (*n* = 236), and Canada (*n* = 199) (Fig. 2). According to the data presented in Table 1, a total of 160 organizations were actively engaged in rehabilitation research pertaining to the COVID pandemic. Nevertheless, a few institutions have emerged as prominent examples, including Harvard University (*n* = 64) in the USA, the University of Toronto (*n* = 62) in Canada, and the University of Milan (*n* = 52) in Italy. Approximately 43 universities have generated more than 30 research documents, while 27 universities have surpassed a threshold of 20 research documents. The International Journal of Environmental Research And Public Health (*n* = 151) is the leading source followed by the BMJ Open (*n* = 71), Plos One (*n* = 57), and the American Journal of Physical Medicine And Rehabilitation (*n* = 51).

**Fig. 3:**
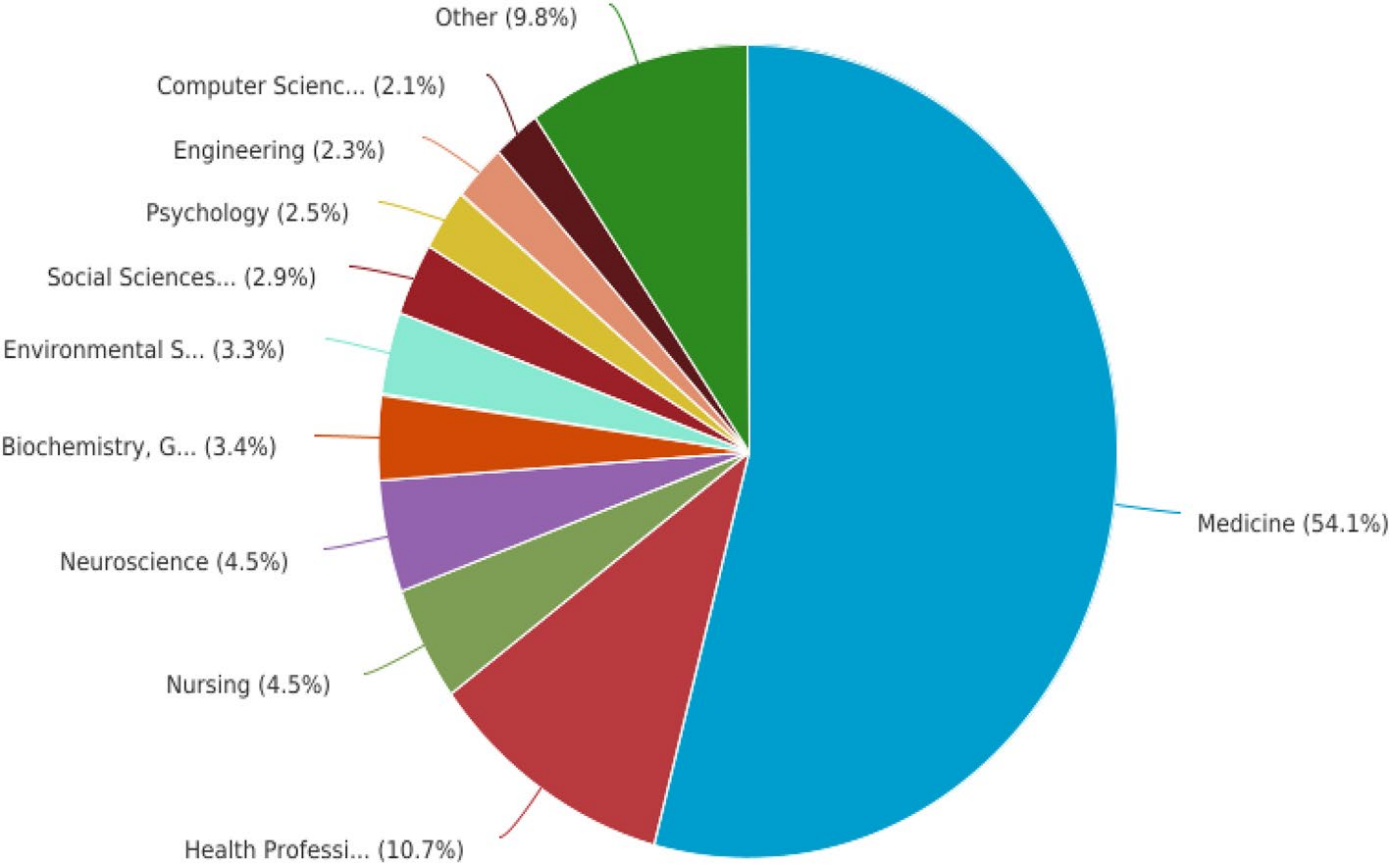
Distribution of documents per subject area related to the COV-REH. The subject areas were Medicine, Health Professions, Nursing, Neuroscience, Biochemistry, Genetics and Molecular Biology, Environmental Science, Social Sciences, Psychology, Engineering, Computer Science, Immunology and Microbiology, Multidisciplinary, Pharmacology, Toxicology and Pharmaceutics, Materials Science, Chemical Engineering, Physics and Astronomy, Agricultural and Biological Sciences, Arts and Humanities, Dentistry, Chemistry, Business, Management and Accounting, Mathematics, Energy, Earth and Planetary Sciences, Veterinary, Economics, Econometrics and Finance, and Decision Sciences

The knowledge framework of COV-REH was established through the involvement of twenty-seven scientific and research disciplines, suggesting that COV-REH exhibits numerous intersections within professional and academic domains (Fig. 3). The proportion of scholars in the field of medicine who participated in this study was approximately 54.1% (Fig. 3). This was followed by the Health Professions (10.7%), Nursing (4.5%), and Neuroscience (4.5%). Additional information is depicted in Fig. 3. The allocation of research areas presented herein is derived from the Scopus database.

### Social analysis: co-authorship

**Fig. 4:**
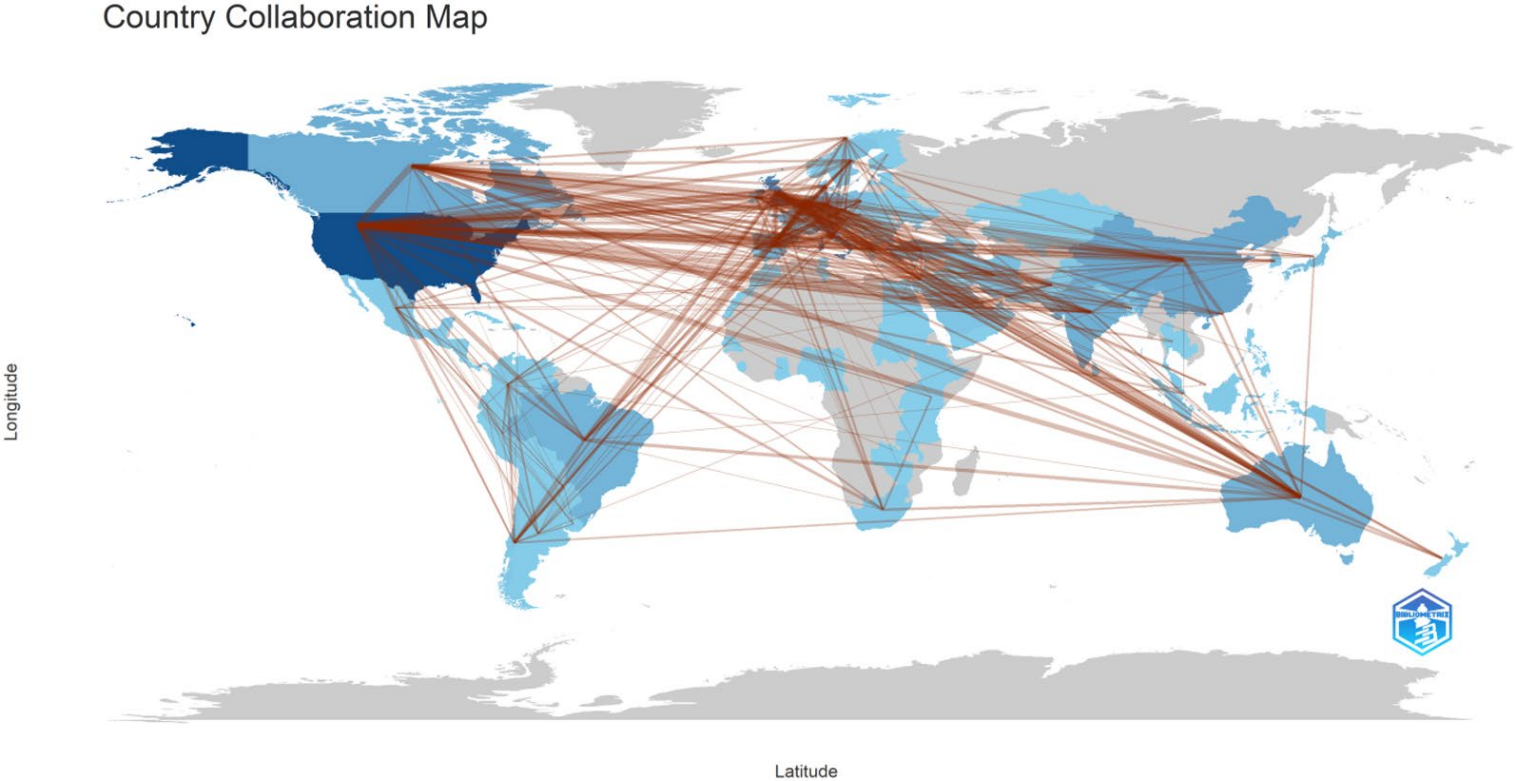
Mapping of international collaboration among countries. Mapping was performed using the Bibliometrix application

In this study, 108 of the documents were authored by a single researcher. The average number of researchers per document is 7.47. According to Fig. 4, the USA, UK, Italy, Canada, and China, achieved the highest number of co-authored documents (420, 399, 233, 153, and 62, respectively). The UK cooperated with 74 countries worldwide to conduct research related to COV-REH, followed by the USA (*n* = 70), then Italy (*n* = 54), Canada (*n* = 53), and China (*n* = 53), respectively. Zambia, Yemen, Sri Lanka, Qatar, Morocco, Kuwait, Hungary, Ethiopia, El Salvador, and Bangladesh were identified as countries that exhibited limited cooperation with other nations, as evidenced by their research output, which was predominantly represented by a maximum of one document. In addition to delineating collaborative research endeavors in nations, it is imperative to consider other bibliographic elements such as academic institutions. The IRCCS Galeazzi Orthopedic Institute, Italy, is the most collaborative organization.

**Fig. 5:**
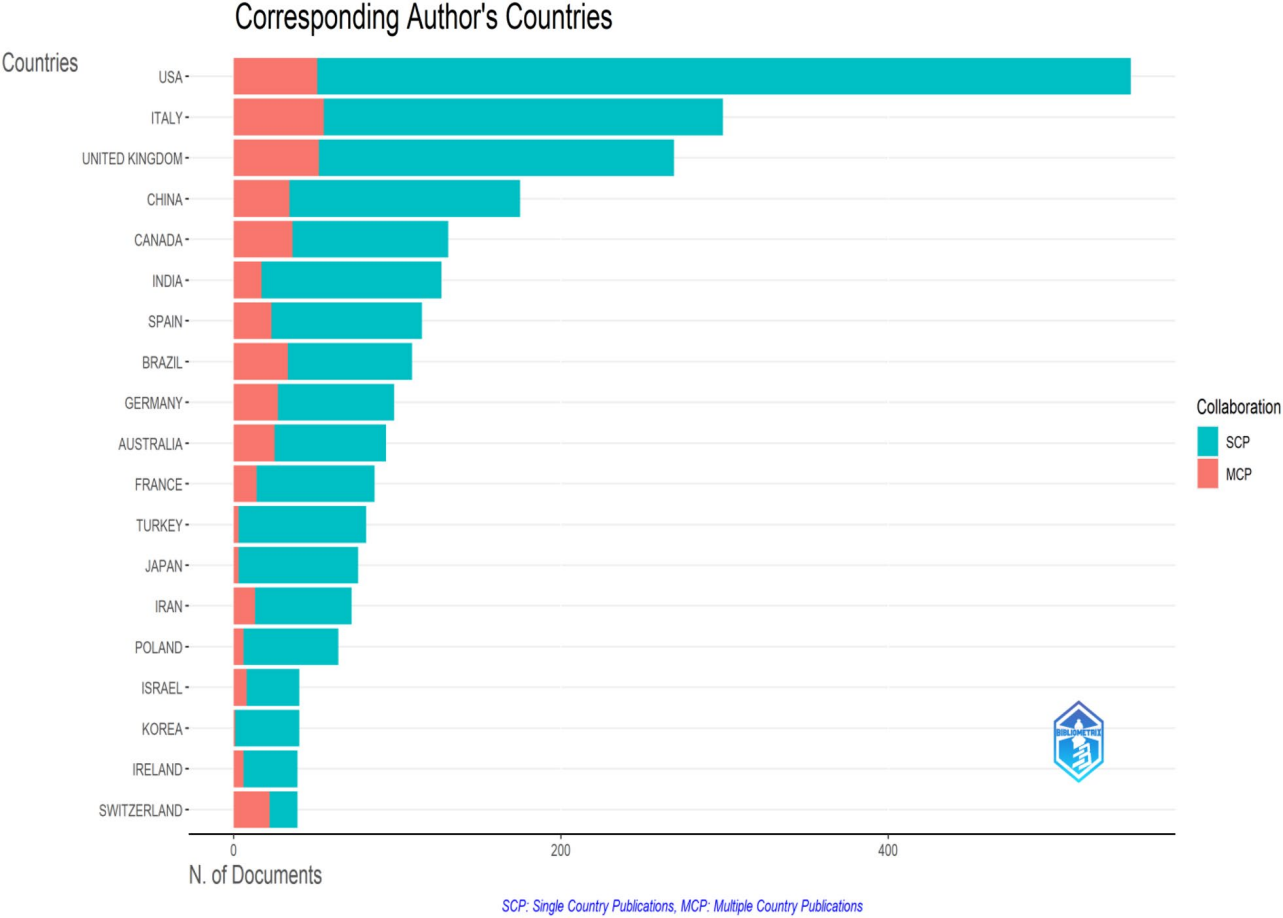
Country of the corresponding author. Collaboration between countries (SCP) and within countries (MCP) from 2020 to 2023. The figure was generated using the BibTex file in the Bibliometrix application

Figure 5 depicts the distribution of countries responsible for the corresponding authorship of a publication, categorized by country. The USA holds the highest position among the top three countries, with a total of 497 research papers. Italy ranks second with a total of 244 published articles, which prominently feature the corresponding authors of Italian origin. The UK occupies the third position producing a total of 217 papers. The nations exhibiting the most pronounced levels of international collaboration include the USA, Italy, the UK, China, Canada, India, Spain, Brazil, Germany, Australia, France, Turkey, Japan, and Iran. It is noteworthy that despite the USA having the highest number of corresponding authors, there is a need for increased national collaboration. Korea exhibits a comparable trend, wherein there is notable scientific output but a deficiency in intra-collaborative efforts.

### Impactfil research: citation analysis

The objective of this study was to employ these methodologies in the context of the COV-REH domain and gain insight into citation patterns and associated attributes. The BMJ, Thorax, European Journal of Physical and Rehabilitation Medicine, Nature Medicine, and International Journal of Environmental Research and Public Health are among the most frequently cited sources in the context of COV-REH research, with citation counts of 1394, 1004, 967, 956, and 852, respectively. The USA (*n* = 10,337), the UK (*n* = 9219), China (*n* = 5332), Italy (*n* = 4978), France (*n* = 3347), Canada (*n* = 2458), and Sweden (*n* = 2113) were the most frequently cited countries. According to the citations and references in the bibliographies of the authors’ works, the USA plays a key role in analyzing their choice of 840 documents.

When examining the trend between the number of citations and the quantity of research documents generated by a singular institution, it becomes apparent that there is a disparity between these two metrics. An illustration of this can be seen in the case of Zoe Global, a company based in the UK, which achieved the highest ranking in terms of citation count. Remarkably, this was achieved by using only two documents, resulting in an impressive count of 956 citations. The subsequent entities in the sequence were West Hertfordshire Hospitals NHS Trust, UK; West Hertfordshire Respiratory Service-Central London Community Healthcare; Veterans Affairs Center for Clinical Management Research, USA; and UCL Respiratory, University College London, UK, all of which have garnered over 400 citations each. When establishing a criterion of five or more documents for a single organization, it was observed that IRCCS Istituto Ortopedico Galeazzi in Italy ranked first in terms of citations, with a total of 598 citations and 14 documents. Following closely is the University of Queensland in the UK, with 332 citations and seven documents, and subsequently Imperial College London, also in the UK, with 285 citations and seven documents.

**Fig. 6:**
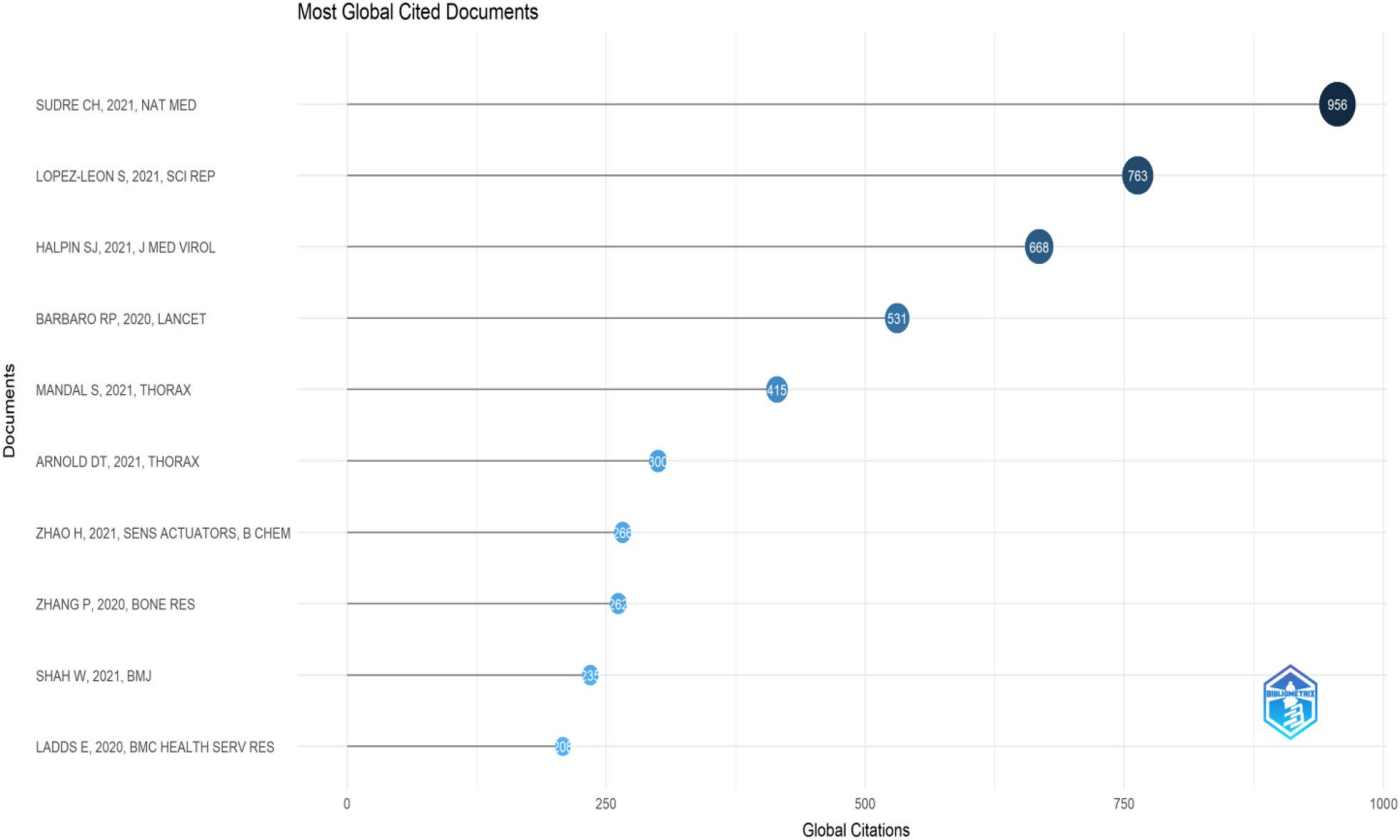
Global citation of documents

Figure 6 presents data regarding the aggregate number of citations received by various papers, their annual citation rate, and the normalized total citations. Presented below is an analysis of the data: Of the papers provided, “SUDRE CH, 2021, NAT MED” exhibits the highest cumulative citations, amounting to 956. This substantial figure underscores the notable influence of this study on the respective fields. The citation rate of 478 per year indicates continuous acknowledgment and impact, as supported by scholarly literature. Furthermore, in the context of normalized total citations, which account for the citation rate in relation to the average within the dataset, this study demonstrated a noteworthy value of 69.90, indicating its exceptional performance. Additional notable papers in the field of study encompass “LOPEZ-LEON S, 2021, SCI REP” which has garnered a total of 763 citations and exhibits a citation rate of 381.5 per annum. Furthermore, “HALPIN SJ, 2021, J MED VIROL” has amassed 668 citations in total, with a citation rate of 334 per year. Both studies have demonstrated a significant impact and recognition within their respective academic disciplines. Moreover, scholarly articles such as “BARBARO RP, 2020, LANCET,” “MANDAL S, 2021, THORAX,” and “ARNOLD DT, 2021, THORAX” exhibit notable citation frequencies annually, suggesting their enduring significance and impact. These papers also demonstrated noteworthy normalized total citation scores, thereby underscoring their substantial influence within their respective fields. In contrast, the research documents were evaluated by considering the number of citations, as indicated in Table 2. The analysis revealed that “SUDRE CH, 2021, NAT MED” did not relinquish its position as the first-ranked entity. However, “GREENHALGH T, BMJ, 2020” held second position in the ranking.

**Table 2:** Top-cited articles based on simple citation count

Rank	Title	Year	Source	Citation	Citation average
1	Attributes and predictors of long COVID [37]	2021	Nature Medicine	956	318.67
2	Management of post-acute covid-19 in primary care [38]	2020	The BMJ	779	194.75
3	More than 50 long-term effects of COVID-19: a systematic review and meta-analysis [39]	2021	Scientific Reports	763	254.33
4	Epidemic of COVID-19 in China and associated Psychological Problems [40]	2020	Asian Journal of Psychiatry	733	183.25
5	Stress and psychological distress among SARS survivors 1 year after the outbreak [41]	2007	Canadian Journal of Psychiatry	713	41.94
6	Postdischarge symptoms and rehabilitation needs in survivors of COVID-19 infection: A cross-sectional evaluation [42]	2021	Journal of Medical Virology	668	222.67
7	Persistence and clearance of viral RNA in 2019 novel coronavirus disease rehabilitation patients [43]	2020	Chinese Medical Journal	585	146.25
8	Extracorporeal membrane oxygenation support in COVID-19: an international cohort study of the Extracorporeal Life Support Organization registry [44]	2020	The Lancet	531	132.75
9	Follow-up study of the pulmonary function and related physiological characteristics of COVID-19 survivors three months after recovery [45]	2020	EClinicalMedicine	488	122.00
10	Mental morbidities and chronic fatigue in severe acute respiratory syndrome survivors long-term follow-up [46]	2009	Archives of Internal Medicine	425	28.33

### Mapping and co-occurrence of keywords: lexical analysis

**Fig. 7:**
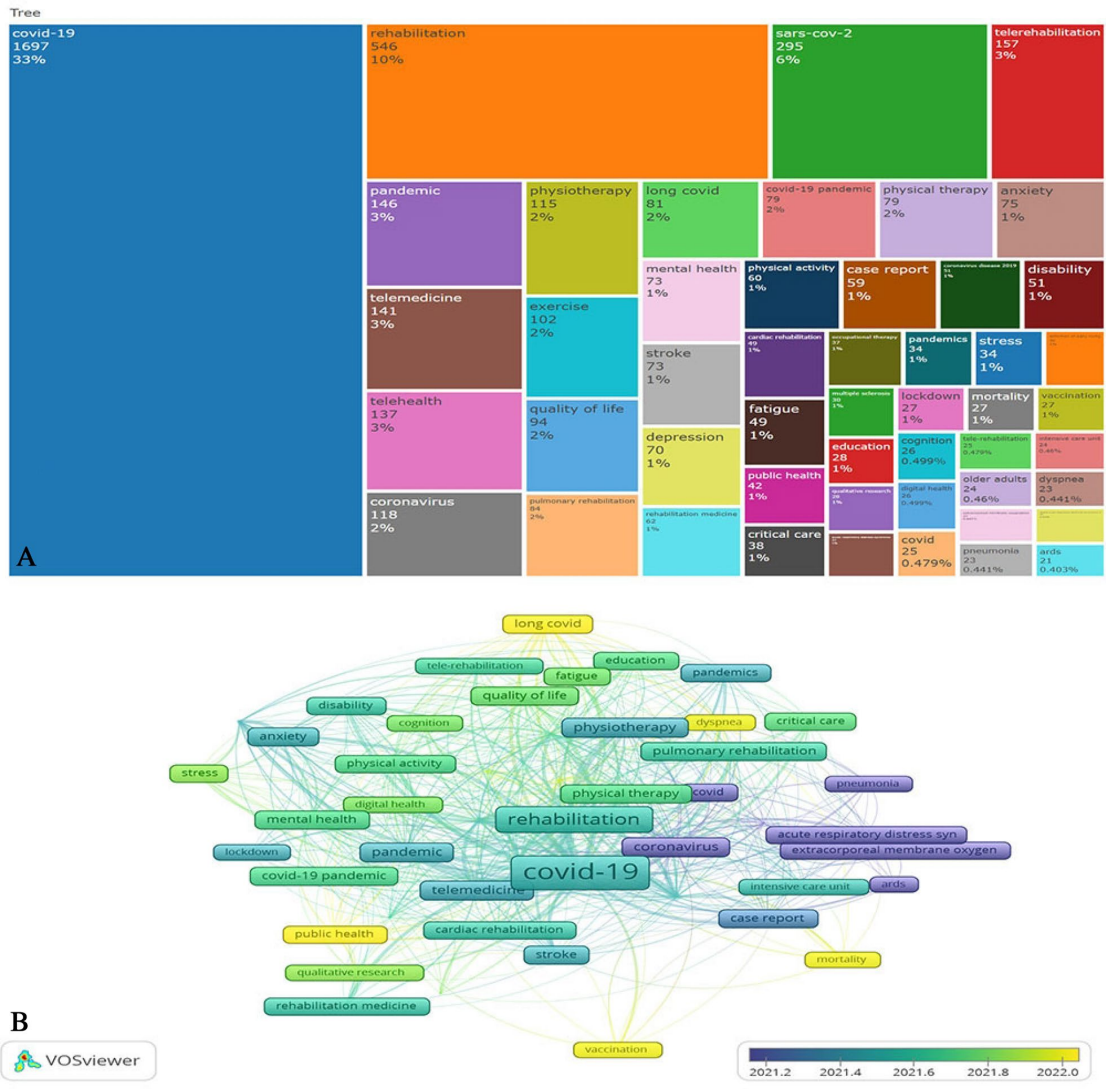
Word tree between 2003 and 2023 (top 50 author keywords). **a** provides an in-depth examination of the research landscape within each time period encompassed by this study (2033–2023). **b** was utilized to ascertain the temporal scope of the latest research advancements (2021–2023) by employing keyword analysis, a distinctive attribute absent in the VOSviewer software. These two figures collectively offer a comprehensive depiction of the research patterns throughout the entire study duration (**a**) and specifically post-2021 (**b**)

The present work demonstrates the effectiveness and usefulness of lexical analysis’s in discovering knowledge components and the structure of the COV-REH field. The most frequent keywords were COVID-19 (freq 1697), rehabilitation (freq 546), sars-cov-2 (freq 295), telerehabilitation (freq 57), pandemic (freq 146), telemedicine (freq 141), telehealth (freq 137), physiotherapy (freq 115), and exercise (freq 102). The word tree between 2003 and 2023 (top 50 author keywords) is shown in Fig. 7a. Figure 7b also depicts the highly frequent keywords, considering their chronological occurrence within the timeframe analyzed in this study. The spectrum of this phenomenon spans from the color violet, which represents the earliest occurrences, to the color yellow, which signifies the most recent events. The most recent keywords in the field of COVID-19 research and healthcare (COV-REH) include “mortality,” “vaccination,” “long-covid,” “dyspnea,” “public health,” “stress,” and “qualitative research.”

#### Thematic map

**Fig. 8:**
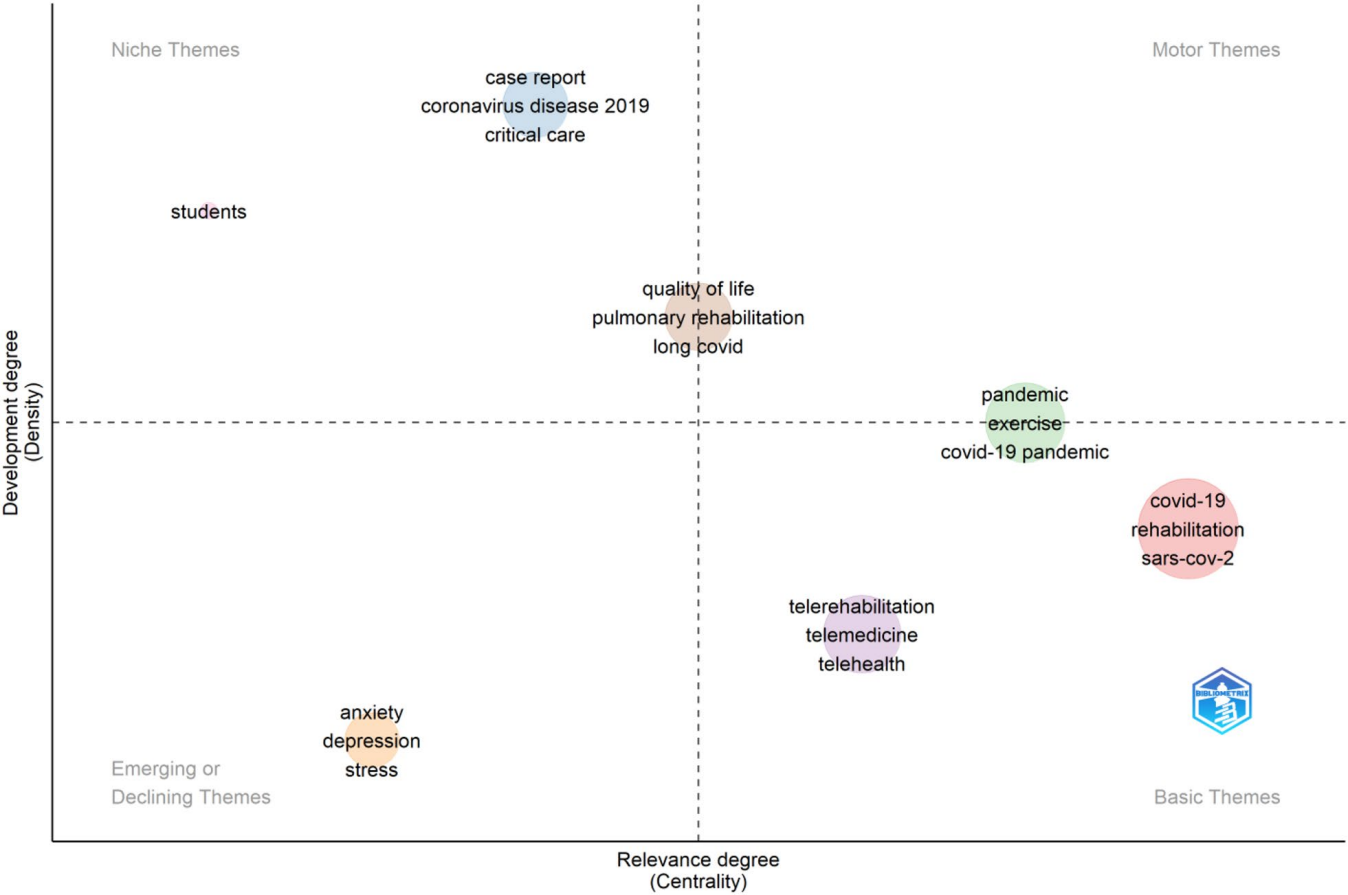
Thematic map by author keywords

The seven research themes covering the entire dataset of these COV-REH studies between 2020 and 2023 are shown in Fig. 8. These are labeled as “covid-19,” “case report,” “pandemic,” “telerehabilitation,” “anxiety,” “quality of life,” and “students.” To facilitate the identification of COV-REH conversations, each cluster was assigned a straightforward label based on the keywords that appeared most frequently within the cluster. Given that these labels serve as the central topics within each cluster, they epitomize the most pivotal subjects in the field of COV-REH research. The magnitude of the spheres corresponds to the quantity of keywords/subjects within a given cluster. The thematic map is detailed as follows:

Two clusters classified as niche themes—“students,” and “case report, corona virus disease 2019, critical care.”

Two clusters for the motor theme—“quality of life, pulmonary rehabilitation, long-covid,” and “pandemic, exercise, covid-19 pandemic.” These two clusters exhibited transthematic behavior.

Two clusters of basic themes—“covid, rehabilitation, sars-cov-2,” and “telerehabilitation, telemedicine, telehealth.”

One cluster is classified as emerging or declining themes—“anxiety, depression, stress.”

Additionally, a categorization of themes was conducted in the field of COV-REH, including basic, motor, niche, and emerging/declining themes. By examining the progression of themes over time, a more nuanced understanding of the conceptual framework within the field can be developed. Table 3 presents the keywords found in each cluster along with their respective frequencies. For instance, the cluster labeled “telerehabilitation” encompasses keywords such as telemedicine, telehealth, physiotherapy, physical therapy, occupational therapy, education, digital health, children, pain, cerebral palsy, and qualitative, with a frequency count of 16. These keywords were grouped together in this cluster because of their consistent appearance in relevant articles. This implies that it is common practice among authors to examine these keywords in conjunction with one another.

**Table 3:** Keywords within the seven clusters and their frequency

No.	Cluster label	Author’s keywords (Frequency)
1	Covid-19	Covid-19 (1681), Rehabilitation (543), sars-cov-2 (295), Coronavirus (118), rehabilitation medicine (60), public health (42), Pandemics (34), activities of daily living (30), qualitative research (28), Vaccination (27), Covid (24), tele-rehabilitation (25), Epidemiology (21), Frailty (19), inpatient rehabilitation (18), coronavirus infections (16), Infection (16), physical therapy modalities (16)
2	Case Report	case report (59), coronavirus disease 2019 (51), critical care (38), acute respiratory distress syndrome (27), Mortality (27), extracorporeal membrane oxygenation (23), intensive care unit (23), Pneumonia (23), severe acute respiratory syndrome coronavirus 2 (22), Ards (21), mechanical ventilation (21), critical illness (20), intensive care (18)
3	Pandemic	Pandemic (146), Exercise (102), covid-19 pandemic (78), mental health (73), Stroke (73), physical activity (60), Disability (51), cardiac rehabilitation (49), multiple sclerosis (30), Lockdown (27), Cognition (26), older adults (24), Outcome (21), virtual reality (21), Employment (18), Prevention (18), Survey (18), Healthcare (16), Nursing (16)
4	Telerehabilitation	Telerehabilitation (157), Telemedicine (141), Telehealth (137), physiotherapy (115), physical therapy (79), occupational therapy (37), Education (28), digital health (26), Children (19), Pain (19), cerebral palsy (18), Qualitative (16)
5	Anxiety	Anxiety (73), Depression (70), Stress (34), Resilience (18)
6	Quality of life	quality of life (94), pulmonary rehabilitation (84), long covid (81), Fatigue (49), Dyspnea (23), post-covid syndrome (21), Recovery (21), post-covid-19 (19), post-covid-19 syndrome (19), coronavirus disease (16)
7	Students	Students (21)

#### Thematic evolution

**Fig. 9:**
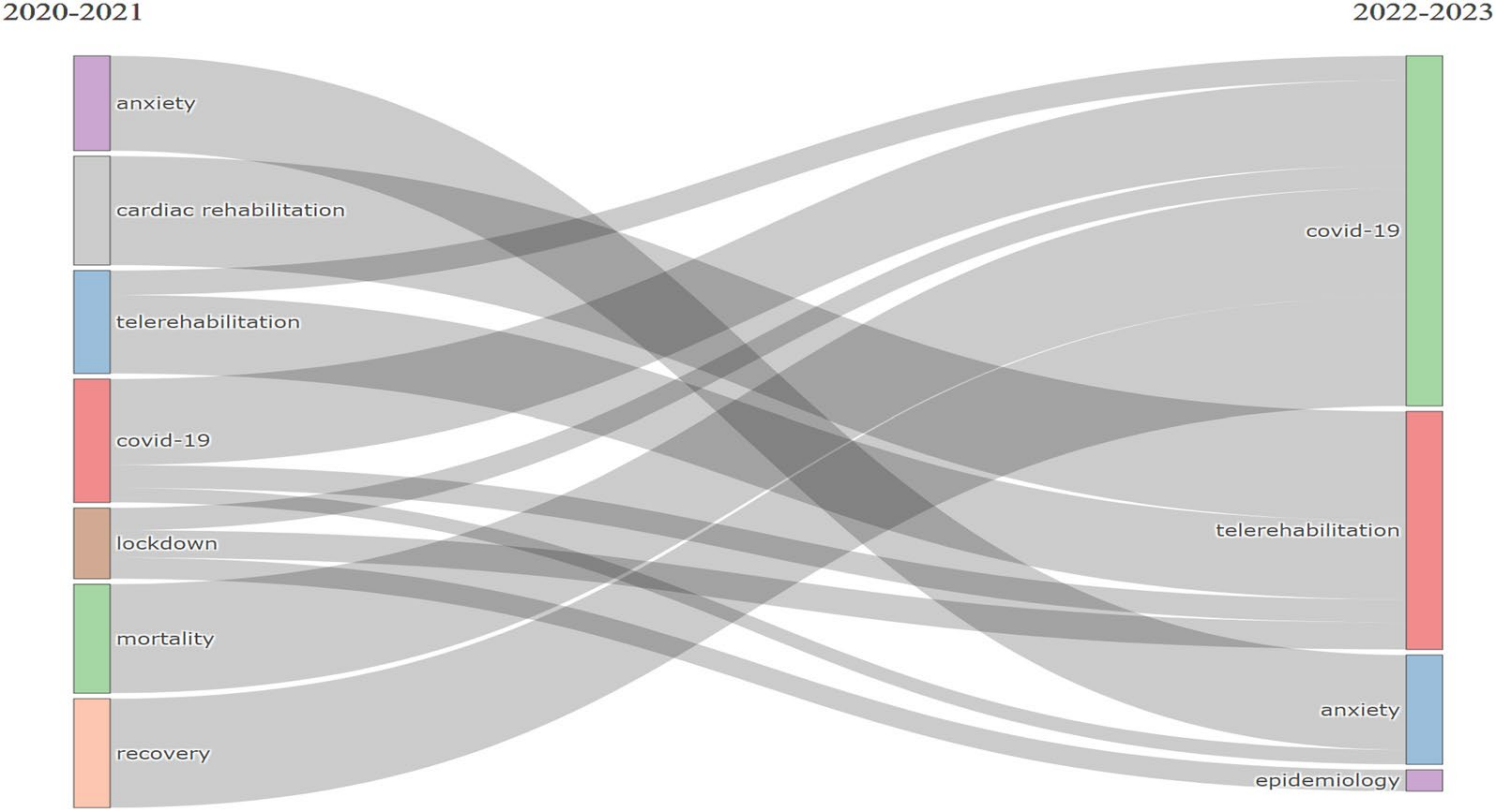
Thematic evolution by author keywords

The progression of the research themes, clusters, or subjects over two time periods (2020–2021 and 2022–2023) is depicted in Fig. 9. Despite evolution and diversification the research themes over time, the fundamental concepts persisted. Throughout the whole COV-REH investigations, the clusters “anxiety,” “telerehabilitation,” and “covid-19” were the top three research themes. Despite the fact that the image concentrates on the evolution of important subjects, core development may be seen between the two time slices. A component of “telerehabilitation” is now the cardiac rehabilitation cluster. Three clusters make up the “Lockdown” topic: “telerehabilitation,” “COVID-19,” and “epidemiology.” Anxiety and “covid-19” were created when the “anxiety” cluster united. The “epidemiology” cluster is derived from the “lockdown” cluster from the standpoint of new clusters in the timeframe 2020–2023. In conclusion, Fig. 9 depicts the overall development of the subjects and fundamental development over two time slices. Between the two time slices, clusters were divided and combined. From a few study themes in the 2020–2021 time slice, a new cluster emerged. It is noteworthy that “epidemiology” emerged as a new research area in the 2020–2023 time frame.

#### Trending topics

**Fig. 10:**
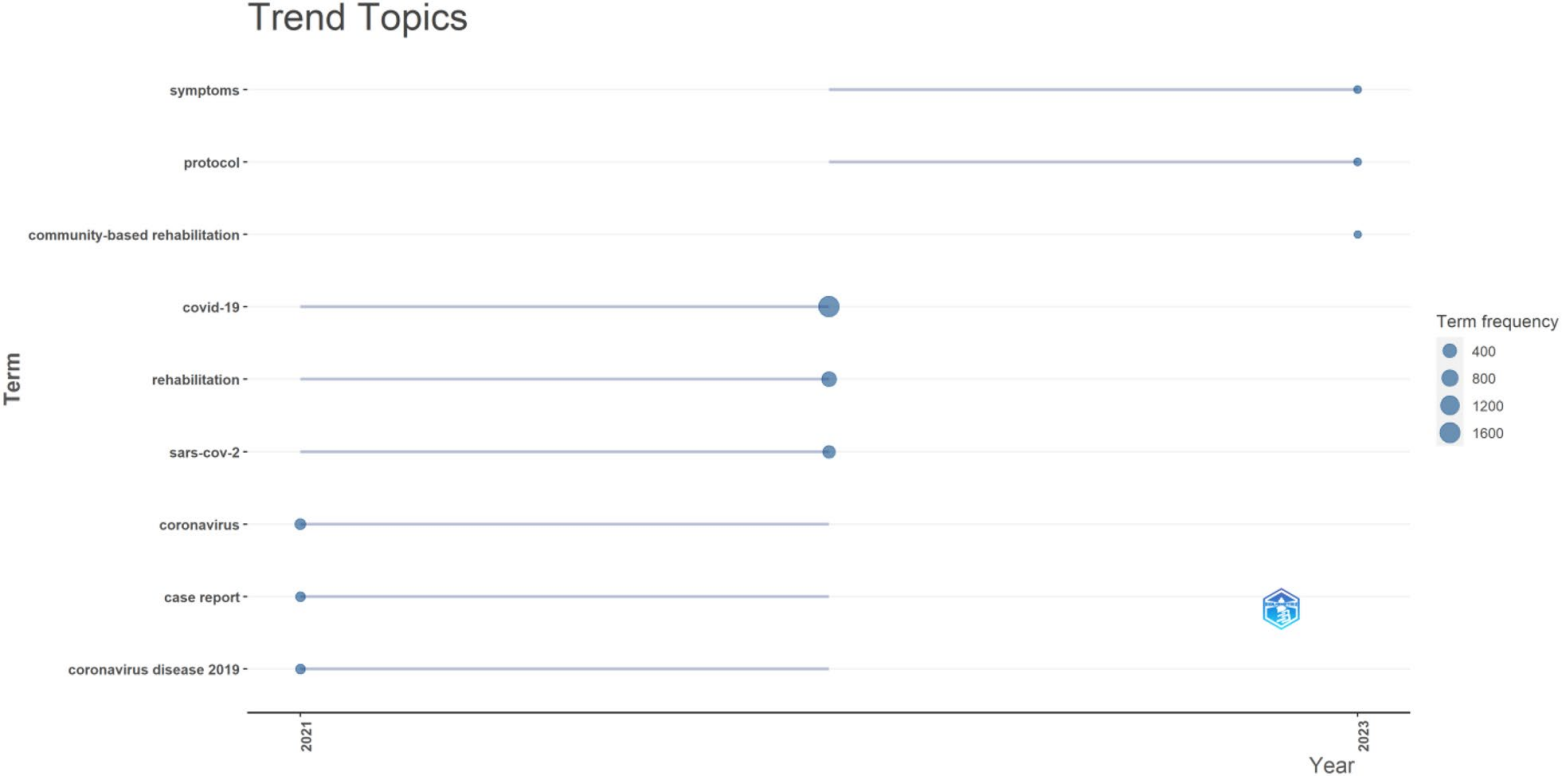
The trending topics in COV-REH

As shown in Fig. 10, the trending topics in COV-REH are “coronavirus,” “symptoms,” “case report,” “rehabilitation,” “rehabilitation,” “protocol,” and “community-based rehabilitation.”

## Discussion

To date, no comprehensive bibliometric investigation has been undertaken in the domain of COV-REH. Particularly, in the aftermath of the COVID-19 pandemic, there has been a notable absence of prospective avenues for future research. Unfortunately, despite its crucial involvement in the COV-REH revolution, a comprehensive quantitative study investigating the significance of COV-REH has not yet been undertaken. The primary objective of this research was to examine the thematic progression, identify emerging topics, and propose potential avenues for future research in this specific field of inquiry. From 2003 to 2023, a total of 3764 original research documents pertaining to COV-REH were produced. The present investigation was motivated to exclusively gather original research as they are regarded as the primary source of knowledge pertaining to any given field. Review papers and books often incorporate the existing knowledge generated by scientific papers, potentially inflating the outcomes of bibliometric studies. The annual growth rate has reached 53.73%. This finding aligns with prior research that has demonstrated the progression of studies pertaining to COVID-19 [47, 48]. The volume of research in the years 2020–2023 is approximately 99. The observed increase in numbers can be attributed to the onset of the COVID-19 pandemic in 2019 [1]. This pertains to the domain of rehabilitation research concerning individuals affected by COVID-19, which has experienced significant growth since the onset of the year 2020 [14, 18, 49–51].

The areas of interest of top-producing scholars focused on pulmonary rehabilitation, integrated rehabilitation pathway, telerehabilitation, multidisciplinary telerehabilitation, and the neuropsychological characteristics exhibited by individuals during their recovery from COVID-19 [13, 16, 31–36, 52–58]. Italy is the country of origin for four out of the five leading scientists whose research has been published in the COV-REH. Italy proposed an evidence-based report outlining an integrated rehabilitative approach for individuals with COVID-19. This approach involves the collaboration of a multidisciplinary and multi-professional team that offers a range of interventions targeting neuromuscular, cardiac, respiratory, and swallowing functions. Additionally, psychological support is provided to enhance the overall well-being and quality of life of patients [9].

The successful pursuit of research in any given field, whether conducted independently or multidisciplinary, relies on a solid understanding of the cognitive content and academic aspects. This enables scientists to effectively investigate various topics by assembling interdisciplinary teams, as needed [59]. Twenty-seven scientific and research disciplines contributed to COV-REH’s knowledge framework, suggesting many professional and academic intersections. About 54.1% of medical scholars participated (Fig. 3). Then came Health Professions (10.7%), Nursing (4.5%), and Neuroscience (4.5%). Previous studies encompassed diverse cohorts of patients, yielding a prevailing consensus that the implementation of multidisciplinary rehabilitation team care significantly enhances the efficacy of rehabilitation interventions [9, 54, 60, 61].

The twentieth century witnessed a notable increase in the magnitude and significance of scientific collaboration, which greatly influenced the process of knowledge production. Science has historically been a collaborative endeavor, as scientists have had to exchange ideas and seek validation from their peers to establish the credibility of their scientific discoveries. However, a multitude of social, economic, technological, and cognitive transformations have led to an unparalleled degree of research collaboration [59]. The findings of the current study revealed that the average number of researchers per document is 7.47. In comparison to other scientometric research, the rate of international co-authorships is 18.8%, showing a high level of scholarly collaboration [62]. In addition to delineating collaborative research endeavors in nations, it is imperative to consider other bibliographic elements such as academic institutions. The IRCCS Galeazzi Orthopedic Institute, Italy, is the most collaborative organization. The IRCCS Institute was the major trauma center in Italy during the pandemic [63]. Owing to the nascent state of clinical experience and research during the initial stages of the pandemic, Italian institutes have substantially contributed to the body of knowledge regarding COVID-19.

In accordance with scholarly conventions, the primary author, who assumes responsibility for submitting the article to the editor of the journal and managing all correspondence, typically includes their email address on the initial page of the article. This serves as a means of communication for fellow researchers seeking to establish contact [64]. The USA holds the highest position among the top three countries, with a total of 497 research papers. Recent analyses of the escalating trend in research collaboration posit that it is primarily propelled by the burgeoning population of scientists seeking funding. This phenomenon has resulted in increased levels of competitive advantage and specialization at the person’s level. In research environments characterized by intense competition, scientists face the imperative of collaborating with colleagues who possess comparable abilities and expertise because of the heightened emphasis on specialization. When individuals seek potential collaborators, they frequently prioritize individuals with elevated levels of prominence and greater scientific productivity, as these individuals possess the ability to facilitate access to limited resources [59, 64–66].

Citation analysis is a pivotal component of the bibliometric assessment of scholarly journals, conferences, institutions, and individual scholars. The emergence of online citation databases has resulted in a rapid increase in their significance and utilization. However, citation analysis poses difficulties when attempting to compare citations across different years, various forms of scholarly communication, or distinct fields of study. The utilization of citation analysis is becoming increasingly significant in the assessment and quantification of the significance of scientists, scientific research, and policy formulation. Simultaneously, there is growing discourse surrounding the usefulness and interpretation of citations [67]. The academic literature on bibliometrics contains a wealth of studies that examine the use of citations across a range of disciplines and fields, encompassing all health-related domains. Bibliometric analyses have been conducted in various fields, subfields, and in specific venues within the COV-REH framework [67]. To the best of our knowledge, this study represents the first comprehensive examination of a diverse range of research on the COV-REH. The USA is the most frequently cited countries. According to the citations and references in the bibliographies of the authors’ works, the USA plays a key role in analyzing their choice of 840 documents. The aforementioned finding can be linked to a number of variables, including geographic location, cultural associations, and linguistic concerns, which have a major impact on preferences toward co-authorship, cross-referencing, and cross-citation [68].

The investigations of the scientific/technical knowledge structure are frequently conducted using two network-based methodologies: co-citation and keyword co-occurrence networks. The primary objective of a co-citation network is to investigate the structure of scientific communication by analyzing the citation links within the literature. Conversely, a keyword co-occurrence network aims to comprehend the knowledge components and structure of a scientific or technical field by examining the connections between keywords within the literature [69]. The present work demonstrates the effectiveness and usefulness of lexical analysis’s in discovering knowledge components and the structure of the COV-REH field. The word tree between 2003 and 2023 (top 50 author keywords) is shown in Fig. 7a. The utilization of this treemap facilitates comprehension of the most significant author keywords within the field of COV-REH. Furthermore, it exemplifies the scientific dialogs that were presumably conducted regarding COV-REH, while demonstrating the interconnections between each keyword hierarchy and the domain of COV-REH. Figure 7b shows the most frequently occurring keywords chronologically in this study. The latest COVID-19 research and healthcare (COV-REH) keywords are “mortality,” “vaccination,” “long-covid,” “dyspnea,” “public health,” “stress,” and “qualitative research.”

In this study, the various themes were observed in the field of COV-REH, including basic, motor, niche, and emerging/declining themes. By examining the progression of themes over time, a more nuanced understanding of the conceptual framework within the field can be developed. The implications of these findings are relevant for both novice scholars and seasoned researchers in the field of COV-REH. The comprehensive understanding of a particular field for young scholars can be achieved through familiarity with influential authors, seminal documents, and important sources. However, experienced scholars can further enhance their knowledge and identify potential areas for future research by employing thematic analysis [70]. Figure 9 shows the development of the research themes, clusters, or topics over two time periods (2020–2021 and 2022–2023). The fundamental ideas persisted despite the research themes’ evolution and diversification over time. The clusters “anxiety,” “telerehabilitation,” and “covid-19” were the top three research themes throughout the entire COV-REH investigations.

The phenomenon of COVID-19, known by various appellations, has consistently remained a prominent subject of discussion throughout the duration of this investigation. The discrepancy in terminology could potentially be identified as a limitation in utilizing keyword analysis as a means of defining cognitive structure. The COVID-19 pandemic has underscored the imperative to enhance rehabilitation services for the most susceptible segments of the population, such as the elderly and individuals with disabilities, to a greater extent than ever. The pandemic has revealed significant deficiencies in healthcare and rehabilitation services in low- and middle-income countries, particularly in impoverished areas, despite the support provided by communities [71, 72]. Chronic SARS-CoV-2 symptoms have major social and economic effects. As the condition spreads, more people may need rehabilitation, straining the healthcare system. Rehabilitation practitioners need clear rules for long-term COVID-19 management. Long-term monitoring of COVID-recovered patient will illuminate “long COVID” care. The term “Long COVID” is used to refer to the ongoing presence of symptoms in individuals who have previously recovered from an infection caused by the SARS-CoV-2 virus [73].

## Conclusion

The pandemic caused by COVID-19 has had a significant global impact, posing significant challenges for rehabilitation services and research initiatives. The COVID-19 pandemic has profoundly affected the delivery of rehabilitation services, resulting to ongoing disruptions in the provision of crucial healthcare services. The COVID-19 pandemic has simultaneously increased the demand for rehabilitation services and research. This demand arises from two distinct groups: those who experience severe manifestations of the illness and require intensive medical care, and those who continue to experience health problems as a result of the viral infection.

Based on the absence of COV-REH-oriented articles in the database searches conducted without a year limitation, it can be inferred that research on COV-REH commenced in 2003. Researchers from Western countries have made significant contributions to the advancement of knowledge in the field of rehabilitation and its research on COVID. The observed phenomenon may be attributed to the enhanced research capacities of academic institutions and the utilization of clinical resources by researchers, medical professionals, and rehabilitation experts to conduct extensive research. Furthermore, this phenomenon facilitated the emergence of Western nations as leaders in terms of both the quantity and caliber of research related to COV-REH. Nevertheless, it is evident that certain nations, including China, have made significant advancements in this area of study, garnering widespread recognition and admiration. The quantitative impact of the aforementioned authors, institutes, and countries, originating from America and Europe, also extends to their direct influence on knowledge formation. Their research is regarded as fundamental to numerous research outputs worldwide. The quantitative and cognitive impacts have rendered these elements more significant in the context of national and international collaboration.

The most frequent keywords provide an initial picture of the research-intensive aspects in this field. The utilization of this treemap facilitates comprehension of the most significant author keywords within the field of COV-REH. The seven research themes (“covid-19,” “case report,” “pandemic,” “telerehabilitation,” “anxiety,” “quality of life,” and “students”) covering the entire dataset of these COV-REH studies between 2020 and 2023. Additionally, a categorization of themes was conducted in the field of COV-REH, including basic, motor, niche, and emerging/declining themes. The progression of research themes, clusters, or subjects is represented over two time periods (2020–2021 and 2022–2023). Despite the evolution and diversification of research themes over time, the fundamental concepts persisted. Throughout the whole COV-REH investigations, the clusters “anxiety,” “telerehabilitation,” and “covid-19” were the top three research themes. The trending topics in COV-REH are “symptoms,” “protocol,” and “community-based rehabilitation.”

This study encourages new lines of inquiry and provides a more complete picture of existing COV-REH investigations, both of which contribute to the literature on the sharing economy. Given the multifaceted nature of COV-REH, studies on the topic should account for a variety of study settings. The bibliometric study laid the groundwork for what is now the most thorough normalcy research on COV-REH imaginable, saving researchers time by highlighting the most frequently occurring topics and publications across time and geography. The recommendations derived from the study highlighted several key areas. First, there is a need for increased research on rehabilitation interventions specific to COVID-19, considering the long-term effects on physical, cognitive, and mental health. Second, collaborative efforts between researchers, healthcare professionals, and policymakers are crucial to developing evidence-based guidelines for effective rehabilitation practices. Third, this study emphasized the importance of integrating technology and telerehabilitation to enhance accessibility and continuity of care. Lastly, future research should explore the impact of socioeconomic factors and health disparities on rehabilitation outcomes in COVID-19. These recommendations aim to inform and guide future research efforts and improve rehabilitation strategies for individuals affected by the pandemic.

## Limitations

The present study encountered certain limitations. A bibliometric study is centered on the cumulative scientific output pertaining to a specific theme or discipline over a specified timeframe. According to the findings, the timeframe under consideration in the COV-REH domain is relatively recent, spanning from 2020 to 2023. Hence, the discipline can be perceived as being in the nascent stage, with its theoretical underpinnings not yet fully established. An additional constraint arises from the potential omission of certain studies in this research, as a result of the inclusion and exclusion criteria set forth by the authors. Additionally, this study was dependent on a singular database, potentially resulting in the omission of bibliographic data that could have provided additional information. Similarly, documents written in languages other than those referred to are not encompassed.

## Data Availability

Data for the current study will be available upon reasonable request from the principal investigator or corresponding author.

## Abbreviations

COVID-19: Coronavirus disease
COV-REH: Rehabilitation and COVID-19
MeSH: Medical Subject Headings
